# Magnetic Resonance Guided Radiation Therapy for Pancreatic Adenocarcinoma, Advantages, Challenges, Current Approaches, and Future Directions

**DOI:** 10.3389/fonc.2021.628155

**Published:** 2021-05-11

**Authors:** William A. Hall, Christina Small, Eric Paulson, Eugene J. Koay, Christopher Crane, Martijn Intven, Lois A. Daamen, Gert J. Meijer, Hanne D. Heerkens, Michael Bassetti, Stephen A. Rosenberg, Katharine Aitken, Sten Myrehaug, Laura A. Dawson, Percy Lee, Cihan Gani, Michael David Chuong, Parag J. Parikh, Beth A. Erickson

**Affiliations:** ^1^ Department of Radiation Oncology, Medical College of Wisconsin, Milwaukee, WI, United States; ^2^ Division of Radiation Oncology, University of Texas MD Anderson Cancer Center, Houston, TX, United States; ^3^ Department of Radiation Oncology, Memorial Sloan Kettering Cancer Center, New York, NY, United States; ^4^ Department of Radiation Oncology, University Medical Center Utrecht, Utrecht, Netherlands; ^5^ Department of Radiation Oncology, University of Wisconsin-Madison, Madison, WI, United States; ^6^ Department of Radiation Oncology, Moffitt Cancer Center, Tampa, FL, United States; ^7^ Department of Radiation Oncology, Royal Marsden NHS Foundation Trust, London, United Kingdom; ^8^ Odette Cancer Centre, Sunnybrook Health Sciences Centre, Toronto, ON, Canada; ^9^ Princess Margaret Cancer Centre, University Health Network, Toronto, ON, Canada; ^10^ Department of Radiation Oncology, Faculty of Medicine, University of Tübingen, Tübingen, Germany; ^11^ Miami Cancer Institute, Baptist Hospital of Miami, Miami, FL, United States; ^12^ Henry Ford Medical Center, Henry Ford Health System, Detroit, MI, United States

**Keywords:** MRI guidance, pancreatic image–guided RT, pancreatic cancer and radiation therapy, pancreatic cancer, MR-guided RT, MR-guided radiation therapy

## Abstract

**Introduction:**

Pancreatic adenocarcinoma (PAC) has some of the worst treatment outcomes for any solid tumor. PAC creates substantial difficulty for effective treatment with traditional RT delivery strategies primarily secondary to its location and limited visualization using CT. Several of these challenges are uniquely addressed with MR-guided RT. We sought to summarize and place into context the currently available literature on MR-guided RT specifically for PAC.

**Methods:**

A literature search was conducted to identify manuscript publications since September 2014 that specifically used MR-guided RT for the treatment of PAC. Clinical outcomes of these series are summarized, discussed, and placed into the context of the existing pancreatic literature. Multiple international experts were involved to optimally contextualize these publications.

**Results:**

Over 300 manuscripts were reviewed. A total of 6 clinical outcomes publications were identified that have treated patients with PAC using MR guidance. Successes, challenges, and future directions for this technology are evident in these publications. MR-guided RT holds theoretical promise for the treatment of patients with PAC. As with any new technology, immediate or dramatic clinical improvements associated with its use will take time and experience. There remain no prospective trials, currently publications are limited to small retrospective experiences. The current level of evidence for MR guidance in PAC is low and requires significant expansion. Future directions and ongoing studies that are currently open and accruing are identified and reviewed.

**Conclusions:**

The potential promise of MR-guided RT for PAC is highlighted, the challenges associated with this novel therapeutic intervention are also reviewed. Outcomes are very early, and will require continued and long term follow up. MR-guided RT should not be viewed in the same fashion as a novel chemotherapeutic agent for which dosing, administration, and toxicity has been established in earlier phase studies. Instead, it should be viewed as a novel procedural intervention which must be robustly tested, refined and practiced before definitive conclusions on the potential benefits or detriments can be determined. The future of MR-guided RT for PAC is highly promising and the potential implications on PAC are substantial.

## Introduction

Pancreatic adenocarcinoma (PAC) has some of the worst treatment outcomes for any solid tumor (1). Median overall survival (OS) remains absolutely dismal for the vast majority of patients afflicted with PAC. It has risen to the fourth leading cause of cancer death in the United States (US), approaching colon and rectal cancer (1). In the next fifteen years, the projected impact of PAC is expected to increase, placing it as one of the top three causes of cancer death by 2030 (2). Radiation therapy (RT) remains controversial in PAC (3). On the one hand, RT is a highly compelling treatment strategy for PAC. Currently RT is successfully applied as a single modality, or in combination with systemic therapy, in curative treatment strategies in most adenocarcinomas and other tumors (4). On the other hand, RT in PAC is challenging due to the proximity of various radiosensitive normal structures like the duodenum, bowel and stomach. Deposition of curative RT doses while sparing the adjacent normal tissues is challenging with conventional RT techniques as the tumor and surrounding structures are highly mobile and difficult to see on CT based imaging. For a long time these limitations have hampered the use of curative RT doses on PAC causing somewhat modest treatment results when using RT in PAC. Recently MR-guided RT has emerged as a potential strategy to improve the therapeutic index of RT (5–8). For a variety of reasons, the MR-guided method seems optimally suited for the treatment of PAC. We sought to summarize and place into context the currently available literature on MR-guided RT for PAC. We highlight the potential promise, but also the challenges associated with this novel therapeutic intervention.

## Methods and Literature Reviewed

A literature search was conducted using PubMed and Google Scholar to identify manuscript publications since September 2014 that specifically used MR-guided RT for the treatment of PAC. The goal of this search was to include manuscripts that describe the treatment of patients using FDA approved MR-guided RT technology. Search terms included: MR guided radiation and pancreatic cancer, MRI and RT and pancreatic cancer, image guided radiation therapy and pancreatic cancer, IGRT and pancreatic cancer. Over 300 search results were individually reviewed and multiple “similar article” links were subsequently referenced and also reviewed. Articles that merely incorporated MRI in the treatment planning process were excluded. Articles considered to be case reports (fewer than 3 patients) were excluded. Articles devoted purely to dosimetric feasibility were also excluded. Clinical outcomes of these series are summarized, discussed, and placed into context of existing pancreatic literature. Attention was given to dose constraints, which are summarized in **Table 1**.

**Table 1:** Select clinical series to have applied MR guided radiation therapy to pancreatic cancer.

Author	N Panc CA	RT Dose/description	Bowel Constraints Applied	Conclusions/Toxicities Reported/Clinical Outcomes	Citation
Bohoudi et al. (9)	10	40 Gy in 5, max doses up to 50 Gy in 5, tumor + 5 mm margin	*Duodenum, Stomach, Small Bowel:* V33Gy < 1 cm^3^ V25Gy < 20 cm^3^	• Clinicians can review and adjust contours within 3 cm from the PTV, both feasible and safe • Faster treatment planning strategy is discussed	(9)
Henke et al. (10)	5/20	50 Gy in 5, goal of 95% coverage by 95% prescription dose, tumor + 5 mm margin	*Stomach Max:* V33 ≤ 0.5 cm^3^ *Duodenum Max:* V35 ≤ 0.5 cm^3^ *Small Bowel Max:* V30 ≤ 0.5 cm^3^ *Large Bowel Max: V35* ≤ 0.5 cm^3^	• SMART is clinically deliverable and safe • Very low rate of toxicity	(10)
Rudra et al. (11)	44	40-55 Gy in 25-28 fractions (n=13) 30-35 Gy in 5 fractions (n=6) 40-52 Gy in 5 fractions (n=16) 50-67.5 Gy in 10-15 fractions (n=9)	Range of institutional constraints included in supplement	• High dose (BED_10_ > 70) had improved 2 year overall survival, 49% versus 30%, p = 0.03 • Freedom from local failure was 77% in the high dose versus 57% in the standard dose • Grade 3 GI toxicity in 3/44 patients, all in standard dose	(11)
Chuong et al. (12)	35	35-50 Gy in 5 fractions, gross nodes also treated. 120%-130% dosimetric hot spots were included, provided OAR constraints met. 20 patients treated with ENI to celiac, SMA, and SMV to same dose as tumor	*Duodenum, Stomach, Small Bowel:* V35 Gy < 0.5 cm^3^ V40 Gy < 0.03 cm^3^ *Large Bowel:* V38 < 0.5 cm^3^ V43 < 0.03 cm^3^	• Median treatment time 83 min (56–108) • Five patients underwent surgery, 1 CR, 2 NCR • 1 year local control was 87.8% • Median time to local progression 7.4 months • 1 year DMFS was 63.1% • 1 year PFS/median PFS 52.4%/7.9 months • Median OS was 9.8 months (from completion of RT) • Acute grade 3 toxicity 2.9%, Late grade 3 toxicity 2.9%	(12)
Hall WA et al. (13)	3/10	Mostly recurrent PAC, previously treated with RT, patients were given 25-35 Gy in 5 fractions	*Stomach:* Max dose of 34 Gy to 0.03 cm^3^ *Duodenum:* Max point dose of 34 Gy to 0.03 cm^3^, 33 Gy < 1 cm^3^, ideal-V20 < 20 cm^3^, V26.5 < 5 cm^3^ *Small Bowel:* Max point dose of 34 Gy to 0.03 cm^3^, ideal-V20 < 20 cm^3^, V26.5 < 5 cm^3^ *Colon*: Max dose less than 34 Gy to 0.03 cm^3^.	• Feasibility was demonstrated for this cohort using 1.5 Tesla MR Linac • Quantitative MRI can be acquired during treatment without longer table times • Longer term follow up needed for clinical outcomes such as late toxicity, OS, and local control	(13)
Hassanzadeh et al. (14)	44	50 Gy in 5 fractions, goal of 95% coverage by 95% prescription dose	*Esophagus, Duodenum, Small Bowel, Stomach Large Bowel*: V36<0.75 cm^3^ for MR Linac 0.5 cm^3^	• Late grade 3 GI toxicity was 4.6% • Median OS was 15.7 months • One year local control was 84.3%	(14)

PAC, pancreatic adenocarcinoma; n, number of pancreatic cases included; CR, complete response; NCR, near complete response; DMFS, distant metastases free survival; PFS, progression free survival; NR, not reported; SMA, superior mesenteric artery; SMV, superior mesenteric vein; OAR, organs at risk; GI, gastrointestinal.

## Discussion

MR-guided RT holds theoretical promise for the treatment of patients with PAC. As with any new technology, immediate or dramatic clinical improvements associated with its use will likely take time and experience. MR-guided RT should not be viewed in the same fashion as a novel chemotherapeutic agent for which dosing, administration, and toxicity has been established in earlier phase studies. Instead, it should be viewed as a novel procedural intervention which must be robustly tested, refined and practiced before definitive conclusions on the potential benefits or detriments can be determined (15).

### Controversies in the Use of RT for PAC

There are several reasons for the seemingly intangible capacity of RT to present itself as a durable and consistently curative modality for PAC. First, and perhaps most relevant, is the high propensity for PAC to metastasize. When the majority of patients develop distant metastatic disease, the ability for a local modality, such as surgery or RT, to demonstrate meaningful improvements in OS, is difficult. Proof of the benefit of RT could be accomplished, but it would require comparative trials of large numbers of patients who survive long enough to demonstrate the benefit of durable local control. Given that the majority of patients with PAC will die of distant metastatic disease progression such trials are difficult and have not been conducted. Regardless of how optimally local control is achieved, this will have been pursued in vain if a patient dies of distant metastatic disease. Despite this, distant metastatic disease is not realized in all patients with PAC, as one third of patients with PAC will die of predominately local disease progression (16). As systemic therapy has become more effective with both cytotoxic approaches and precision medicine strategies, this percentage will likely increase (17, 18). Maximizing local therapy will therefore become increasingly important for patients with PAC and will potentially lead to better OS in an era of more effective systemic therapies. Local progression causes morbidity, which is difficult to treat. Effective local therapies can reduce symptoms and improve quality of life, both of which RT has been consistently shown to effectively accomplish (19, 20).

### RT Challenges in PAC

PAC creates a trifecta of difficulty for effective treatment with traditional RT delivery strategies. First, is the significant difficulty of visualizing pancreatic tumors using traditional CT-based imaging strategies (21). The boundaries and locations of these tumors are exceptionally difficult to distinguish (18). Pancreas cancers are difficult to define on CT as they are hypo-attenuating with ill-defined borders. Even after contrast delivery, the Hounsfield unit difference between cancer and normal pancreatic tissue are nearly identical. Five to 14% of PACs are often iso-attenuating, blending imperceptibly with the normal pancreatic parenchyma. Second, is the location of pancreatic tumors close to exquisitely radiosensitive normal organs at risk for injury, specifically the small bowel and stomach. Critical is the fact that the small bowel is a “serial” organ at risk. Meaning if even a small portion of this organ is injured, the function of the entire organ is compromised. Clinical consequences of small bowel injury can be dire. The presence of the small bowel intimately associated with pancreatic tumors dramatically limits the ability to deposit meaningful doses of RT. Higher doses of RT have been associated with improvements in both OS and local control (22, 23). Yet, this must be done with exquisite caution for the small bowel in close proximity. Third, is the presence of highly variable, and unpredictable movement of both the primary pancreatic tumors and the adjacent normal organs. This trifecta is difficult to overcome, even with novel strategies using heavy ions, which are also susceptible to the unique challenges presented by PACs (24). Each of these components aggregate to make delivery of curative doses of RT to PACs exceedingly difficult to accomplish. Beyond just the total dose of RT, another currently controversial and challenging area is the optimal treatment volume that should be included. While historic strategies with SBRT included tumor only, there are recently published patterns of recurrence data that suggest the possibility of higher local and regional recurrences around the vasculature associated with focal SBRT including only the tumor (25, 26). Local recurrence along vascular structures, secondary to nearly ubiquitous peri-neural and peri-vascular invasion in PAC, remains a major concern. Historically, treatment volumes with fractionated RT have almost uniformly treated regional vascular structures to reduce this recurrence event. The high rates of regional nodal failure, secondary to peri-neural and peri-vascular spread, should be closely considered by radiation oncologists.

### MR-Guided Radiation Therapy

MR-guided RT is a novel treatment technique that has emerged in the past 5 years and presents promise for a variety of solid tumors. There are two commercially available MRI Linear accelerators (MR-linac) systems including one by ViewRay (ViewRay Inc., Oakwood Village, Ohio) and a second by Elekta (Elekta AB, Stockholm, Sweden) (5). Several review articles have been published on this topic and a detailed overview of MR-guidance is beyond the scope of this article (27–29). In brief, rather than using a CT unit installed within a linear accelerator to localize the position of a tumor and normal organs prior to treatment delivery, a MR-linac combines an MRI device with a linear accelerator. Such a combination enables several capabilities that are uniquely helpful for the treatment of PAC. First, MR-guidance offers improved soft tissue contrast and thereby the ability to distinguish the boundaries of different types of soft tissue. This can include the location of a tumor, small bowel, stomach or vascular structures. Second, is that MR imaging on both commercially available MR linear accelerator devices is enabled when the beam is turned on and actually delivering RT. This results in the ability for normal organ movement to be tracked and monitored during the actual time of RT delivery. Such “real time” organ movement enables intra-treatment monitoring and will ultimately enable advanced dose tracking strategies. In other words, the precise radiation doses that were actually given to tumor and the normal structures will be understood during the actual treatment delivery. Real time imaging will enable entirely novel tracking approaches, previously unappreciated. Third, with MRgRT at each fraction a new treatment plan can be generated based on the actual MRI visualized anatomy. This is especially important for targets in areas were a large interfraction variation is expected like in PAC. Finally, in addition to anatomical imaging, functional and biological MR imaging can be routinely acquired, the meaning of which remains to be defined in most solid tumors. However, there is robust literature in the diagnostic space that many solid tumors exhibit early and clinically meaningful changes on MRI during a course of either chemotherapy or RT (30).

### Rationale for MR-Guidance In PAC Over CT-Based Image Guidance

**Figure 1:**
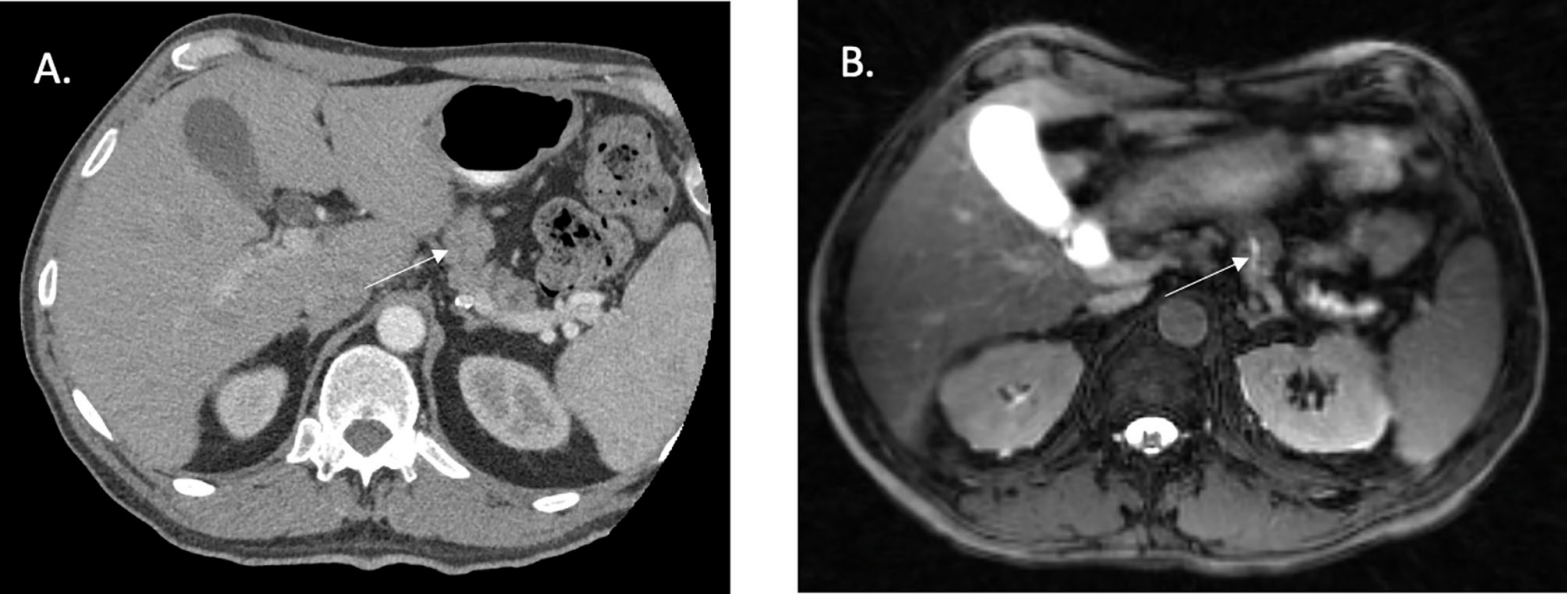
CT simulation and fat suppressed T2/T1 MR images acquired from a 1.5 Tesla MR Linear Accelerator. **(A)** CT Simulation with contrast highlighting difficult to visualize pancreatic body primary tumor. **(B)** Slight improvement in visualization with images from 1.5 Tesla MRL, yet still difficult.

**Figure 2:**
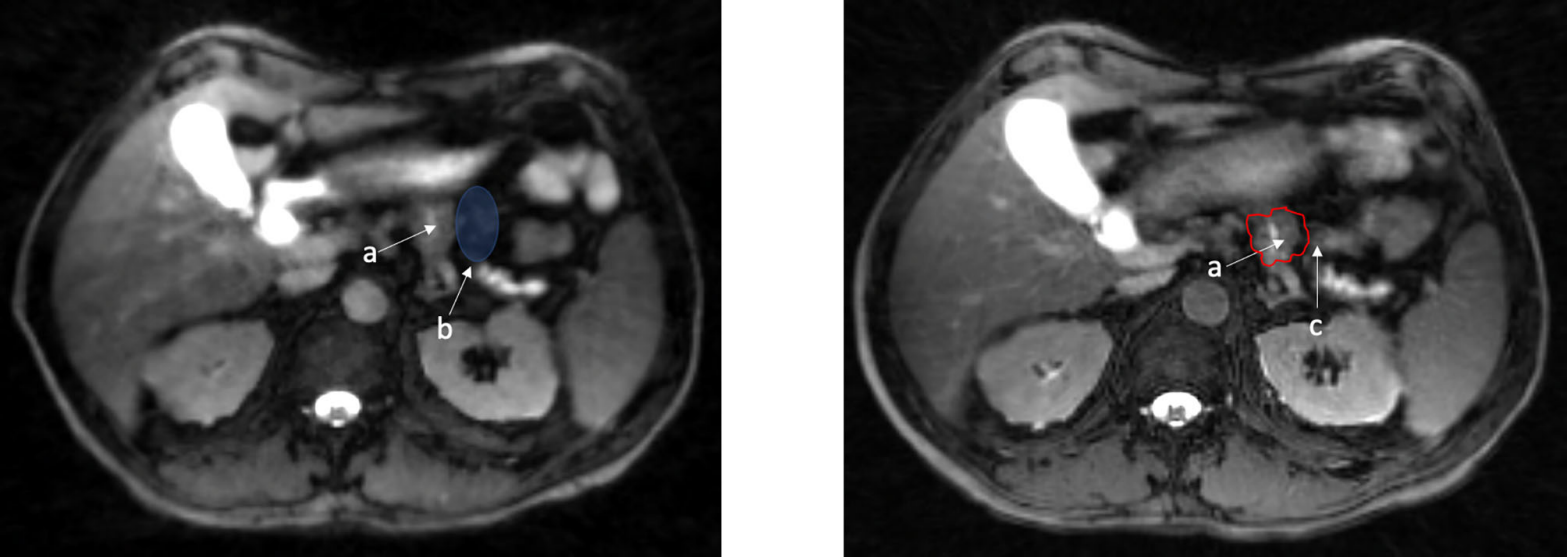
Fat suppressed T2/T1 images acquired on a 1.5 Tesla MR Linac with illustration of a tumor in a close proximity to a potential space that can be occupied by moving small bowel. a. Small biopsy proven pancreatic body tumor. b. Potential space for small bowel to move. c. Example of small bowel movement in close proximity to gross tumor, max dose went from 26 Gy to 35 Gy (red 35 Gy).

MR-guided therapy has recently presented itself as a highly appealing new option for patients with PAC. MR-guidance directly addresses several of the pivotal issues that have existed for decades with CT-based image guidance. First, is the ability to distinguish a tumor from normal pancreatic tissue. An example of a pancreatic tumor on CT simulation is seen in **Figure 1A**, despite a contrast enhanced CT, the ability to accurately identify the edges of many pancreatic tumors is nearly impossible. This is modestly improved with the use of a 1.5 Tesla MR-linac, even without IV contrast, as seen on the MR-linac image in **Figure 1B**. Additional work is needed to highlight the locations and conspicuity of pancreatic tumors. Highlighted in **Figure 2** is that many pancreatic tumors are located in such a position that the movement of small bowel can dramatically impact the dose of RT to those organs. An example of this is seen in the shaded region between **Figure 2**. The presence of bowel in this area changed significantly between fractions, and dosimetrically the recorded versus the observed bowel doses were significantly different. There is almost no question this normal organ movement has dramatically impacted RT dose in a variety of tumor sites, and especially in PAC.

### Existing Series That Have Treated PAC Using MR Guidance

Despite MR-guidance being a relatively novel technological treatment strategy, there are several published retrospective series that have examined the ability of MR-guidance to improve the treatment of PAC. The majority of these published series have used the ViewRay MR-guided linear accelerator system (5), primarily because this has been FDA approved for a longer period of time than the Elekta MR-linac, and consequently accumulated more clinical data. Most of the currently published data is early feasibility work or small retrospective assessments.

One of the earliest clinical experiences examining the use of MR-guidance for the treatment of PAC was published in 2017. Bohoudi et al. describe stereotactic MR-guided adaptive radiation therapy, “SMART”, for the treatment of PAC. In this study, the gross tumor was contoured and a 3 mm planning target volume (PTV) margin was applied. A total dose of 40 Gy in 5 fractions was prescribed, allowing 1% of the PTV to go to 50 Gy (9). This series also presented the feasibility of physicians adjusting the contours of the organs at risk (OAR’s) within 3 cm of the PTV, rather than the entire abdominal cavity. Shortly after this publication, Henke et al. published their experience treating abdominal tumors that included a total of 3 patients with recurrent PAC, along with 2 patients with primary PAC (10). This series also included patients treated for other abdominal tumors such as intra-hepatic cholangiocarcinoma, primary hepatocellular carcinoma, as well as metastatic disease. Conclusions from this series were that treatment with MR-guided RT was safe with low rates of toxicity. A relatively small number of patients with PAC, however, were represented in this series.

There have been three series published in the past two years including 25 patients or greater that have retrospectively assessed local control, toxicity, and OS associated with MR-guided RT in PAC (11, 12, 14). These series start to provide a window into clinical outcomes in patients with PAC treated with MR-guided RT. Important to consider is that MR-guidance is a highly novel treatment strategy, using unique and complex technology. Similar to many other complex oncologic interventions (such as robotic surgery) optimal outcomes will take time to emerge as techniques, methods, and skill sets using this technology develop and expand. While learning curves are well documented for some novel surgical techniques, they remain poorly studied and understood in advanced RT delivery (9, 31).

In the first of these series, Rudra et al. presented the results of 44 patients treated for inoperable PAC. This was a multi-institutional series that was one of the earliest to have aggregated data and presented outcomes focused specifically on high dose RT given with MR-guidance in PAC. Interestingly, OS was improved with the use of high dose (a BED_10_ dose greater than 70 Gy) MR-guided radiation in this series, 49% versus 30%, p = 0.03, with impressive rates of local control (over 75%) without any grade 3 toxicity. Given the retrospective nature of this series, there is the significant possibility of selection bias that must be considered when interpreting this data (11). Hassanzadeh et al. recently published their single institutional data examining patients treated with high dose ablative radiation for PAC (14). Again, high rates of local control, over 80%, with very acceptable rates of GI toxicity were demonstrated. Median OS rates in the series remained relatively similar at 15.7 months, which is similar to non-ablative, conventionally fractionated series from multi-institutional prospective trials. Significant work remains to understand how improved patient selection can contribute to improvements in OS.

Finally, Chuong et al. recently published a retrospective analysis of 35 patients treated using the ViewRay technology (12). They demonstrate excellent rates of local control and low reported rates of toxicity. Again, despite these seemingly strong outcomes, median OS and PFS were relatively similar, compared with other SABR pancreatic series. Important to note is the time point from which follow up data is being measured (from the end of RT versus time of diagnosis). **Table 1** summarizes the existing clinical series to have examined the treatment of PAC using MR-guidance.

## Ongoing Prospective Trials

**Figure 3:**
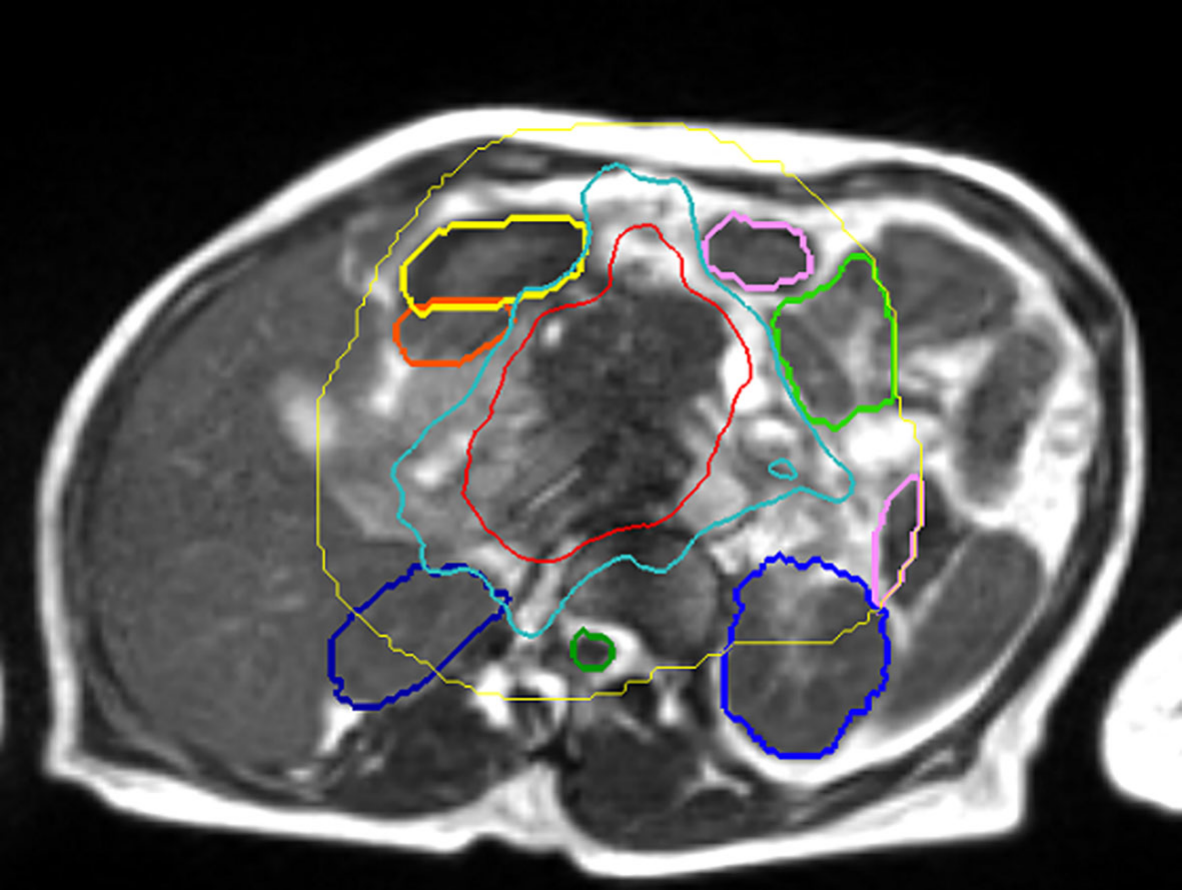
View Ray 0.35 Tesla T2/T1 MR Guided RT. “SMART” patient (NCT03621644) – 50 Gy isodose in red, 33 Gy in cyan. Stomach in yellow, duodenum in orange, small bowel in green, kidney in blue (courtesy of Dr. Parag Parikh).

Prospective research is desperately needed to examine novel RT applications in PAC. While retrospective series provide some framework, they should only be used as tools to design optimal prospective trials. Patient selection, and the potential for bias in retrospective studies is a confounder that can simply never be overcome. There are several ongoing trials that specifically focus on MR-guidance in PAC. The SMART trial is a well-known phase II trial examining the use of MR-guided radiation for locally advanced PAC and is currently accruing (NCT03621644). A total of 133 patients are planned for enrollment into this multi-institutional trial. The primary endpoint of this study is grade 3 or higher GI toxicity within 90 days of completion of RT. Given the relatively modest improvements in outcome over CT-based image guidance associated with MR-guided RT thus far, the SMART trial will ideally set the stage for future randomized trials providing a robust comparison between both CT and MR-guidance based RT modalities. An example of a patient treated on this clinical trial can be seen in **Figure 3**.

A second currently on-going study at Dana Farber Cancer Institute is a phase I/II study involving patients with either PAC, lung cancer, or renal cancer (NCT04115254). Primary endpoint for the phase I portion of the study is delivery success rate for SMART across multiple tumor types.

Finally the MOMENTUM study (NCT04075305) is an ongoing prospective registry that is currently collecting outcomes for patients treated with multiple solid tumors, including PAC using 1.5 Tesla MR-guidance. In this multi-institutional study, consisting of 7 centers with Elekta Unity linear accelerators, patients are being prospectively enrolled and followed for a multitude of outcomes. Patient-reported quality of life along with other detailed clinical outcomes data is being collected, including local recurrence and toxicity events. This will subsequently be used to inform prospective trials comparing MR-guided radiation with CT- based radiation.

## Current Logistical Approaches to Online Adaptive MR Based Image Guidance

A detailed discussion of methods, contouring strategies, and consensus approaches for implementation of online adaptive MR guidance for PAC is beyond the scope of this article. There are some helpful publications on PAC in general (32), not specific to MR-guidance (32). It should be recognized that online adaptive MR-guidance is a highly complex procedure that requires an engaged multi-disciplinary team including radiologists, radiation oncologists, physicists, therapists, and scheduling coordinators. The details of pancreatic tumor dosing and MR image guidance implementation has been the subject of recent publications. Specifically, tumor and normal organ delineation for PAC using MRI has been addressed in two recent review articles (33, 34). Dosing strategies, particularly those that may accomplish ablative dosing, have also been the subject of several recent review articles (35–37). Ablative dosing likely offers a higher probability of local control, and its implementation may be facilitated with online adaptive MR-guidance; but this remains to be conclusively determined. In addition, device specific methods of online adaption that could also be considered have also been published (29). Finally, more practical methods for logistical delivery have been the subject of other recent publications and maybe of use for centers considering implementation of online adaptive MR guidance (13). Each of the clinical outcomes series presented in **Table 1** have associated methods that can be referenced for consideration regarding specific details of treatment strategies that have been applied. In addition, institutional selection criteria as to how patients are chosen for MR guidance methods in the upper abdomen have been previously published (13). In general, collaboration with experts, multi-disciplinary teams, and enrollment into clinical trials (with clear treatment protocols) is an optimal strategy for MR-guided treatment. At this time, the optimal strategy for MR guidance in PAC is still being determined, and clinical trials with detailed methodology is the best strategy for that determination.

## Future Directions

**Figure 4:**
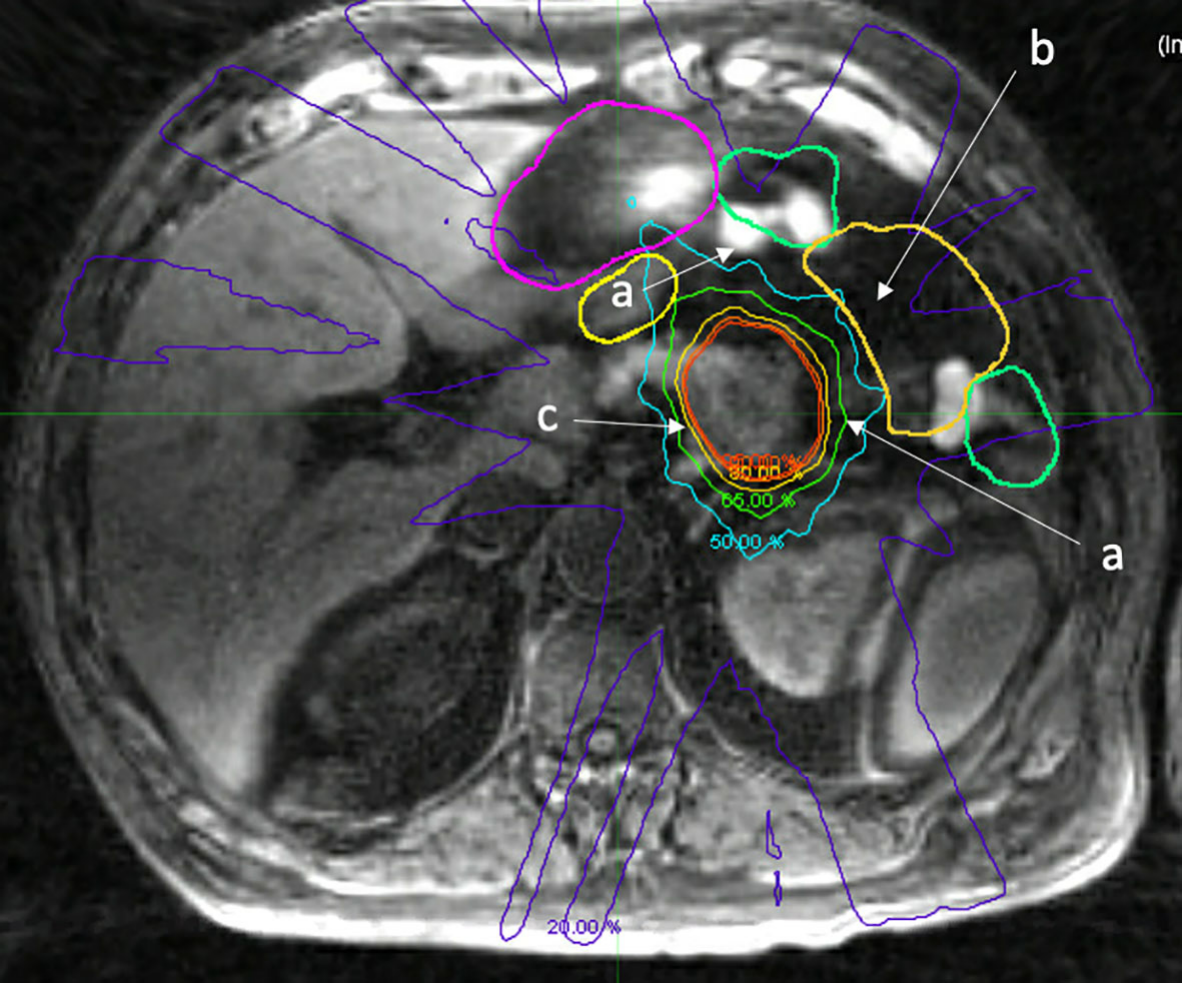
Fat suppressed T1 image acquired on a 1.5 Tesla MR Linac immediately after treatment delivery highlighting normal organ movement during treatment that reflects uncertain dosimetric consequences. a. Movement of small bowel during treatment, differing from adapted contours (green, yellow). b. Void of small bowel that opened during treatment, actual RT dose to small bowel is likely not accurately measured, despite daily adaption. c. Isodose lines highlighting prescription dose with fall off.

The future of RT in PAC is at a critical precipice. Technology is rapidly evolving that will improve capabilities with RT. However, our understanding of how this technology should be optimally applied in PAC is contingent on prospective trial enrollment and detailed clinical outcomes publications. Traditional RT concepts, such as planning risk volumes (PRV’s) accounting for normal organ movement or appropriate PTV expansions, are occasionally questioned for patients being treated with real time MR-guidance. **Figure 4** presents an example of how, despite optimal contour adaption before treatment, normal structures moved during treatment, and the dosimetric consequences of this movement are difficult to quantify and are poorly understood with current technology. Such movement may continue to justify including a PRV and PTV, unless it can be corrected or accounted for with exquisite accuracy in real-time. In theory, real-time treatment plan adaptation as the RT beam is delivering radiation dose could overcome this issue, however the computational time requirement associated with plan re-calculation times and imaging acquisition are currently prohibitive. That being said, it is only a matter of time before this computational power and ability is an immediate reality. This will very likely dramatically shorten treatment times and improve plan quality. There are many additional areas ripe for improvement in the therapeutic ratio in PAC. These include biological imaging-based response assessment (30), artificial intelligence-enabled real time contour adaptation (38), along with novel methods to account for accumulated RT dose to critical local normal structures. The future of highly personalized and adaptive RT in PAC is exceedingly promising, and radiation oncologists must lead the way *via* the education of our surgical and medical oncology colleagues. Novel RT treatment strategies need to be considered. Radiation oncologists must work closely with therapists, and physicists to optimize RT delivery and conduct ground-breaking clinical research. The systematic publication of outcomes is absolutely critical. Finally, randomized trials comparing MR-guidance to CT guidance would be helpful to quantify the magnitude of any benefit.

## Author Contributions

All authors contributed to the article and approved the submitted version.

## Funding

The project described was supported by the National Center for Advancing Translational Sciences, National Institutes of Health, Award Number KL2TR001438. The content is solely the responsibility of the author(s) and does not necessarily represent the official views of the NIH.

## Conflict of Interest

LaD: Licensing agreement with Research, funds paid to institution. SR: Consults for Novocure Gani: University Hospital Tübingen receives financial and technical support including costs for travels and symposia from Elekta AB (Stockholm, Sweden) under a research agreement.

The department of radiation oncology at the Medical College of Wisconsin receives research funding from Elekta AB.

The remaining authors declare that the research was conducted in the absence of any commercial or financial relationships that could be construed as a potential conflict of interest.
